# Health Education as a Means of Addressing Death in the Elderly

**DOI:** 10.3390/ijerph18126652

**Published:** 2021-06-21

**Authors:** Nazaret Martínez-Heredia, Andrés Soriano Díaz, Ana Amaro Agudo, Gracia González-Gijón

**Affiliations:** Department of Pedagogy, University of Granada, 18011 Granada, Spain; asoriano@ugr.es (A.S.D.); anaamaro@ugr.es (A.A.A.); graciag@ugr.es (G.G.-G.)

**Keywords:** elderly adults, health, quality of life, active ageing, well-being, education for death, qualitative

## Abstract

Education for death is an emerging field of study in which health education research and proposals are increasingly being made with the aim of acquiring knowledge and skills to promote positive attitudes towards health and preparation for the end of life. The aim of this study is to find out what experience older people have had with death education and the importance they give to health education. A qualitative methodological design was selected using a semi-structured interview. The survey consisted of interviews with 28 participants from the city of Granada (Spain) aged 61 to 78. This qualitative-descriptive study is based on an analysis of older people’s experience of education and preparation for death throughout their lives. The results show that, in most cases, the only information received was in childhood and always from a religious perspective. Death and health are closely related, so working on death education helps to improve the quality of life of elderly people. Health education offers ways of coping with the end of life through the transmission of values and practices that make it possible to anticipate and resolve situations of instability or anxiety. Facing death naturally and as just another part of life will help to make healthy ageing possible, through educational proposals related to the integral health of elderly people.

## 1. Introduction

Death is a field of knowledge that is beginning to be analysed in a multidisciplinary way. Conceptual, historical and cultural aspects, values and beliefs emerge from its study, transforming it into an event of its own character [1]. Due to the invisibilisation to which it has been subjected by our culture, it has remained hidden, above all, in the imagination of the younger generation.

People, regardless of their age, do not know the circumstances of their death, so fear and uncertainty generate a remarkable set of emotions, including fear and anxiety [2]. In old age, in particular, a series of processes are set in motion, including an increase in thoughts about the proximity and arrival of death, which allows for a clearer appreciation of the fact that life is limited and a greater awareness of mortality [3]. The process of dying is also affected by other factors, including the presence of close deaths of family members or friends and the need for grief processing both for oneself and others [4].

Education is an instrument that can be used for the development of healthy and active ageing, which is why, more specifically, health education for older people is closely linked to well-being and the improvement of quality of life, to address situations that may generate emotional or physical discomfort, such as the issue of death [5]. It is therefore essential to provide training for elderly people, aimed at demystifying the fear of death and making it no longer a taboo subject in our Western system [6]. To be able to offer actions of an educational-palliative nature, it is essential to design training processes that, in accordance with the dimensions of the human being and their need for care, give rise to a comprehensive education that makes it possible for the person to build their own life project [7]. To this must be added the need for education for death, from a transversal perspective, which allows its acceptance as a process and a natural fact, for which it is necessary to prepare oneself to face it as a universal reality. In end-of-life care, we can consider good practice that aimed at achieving adequate objectives, based on promoting the dignity and quality of life of the elderly person. The means for this include comprehensive care for the elderly and family members, optimal control of symptoms, emotional support and adequate communication [8,9,10]. End-of-life care requires training by the team to ensure that the care plan is successful by providing an improvement in the quality of life of the person [11,12].

The main proposals and investigations of Education for death in Spain focus on three major categories, grouped in the spiritual, emotional, social, cultural, religious and formative value of death for evolution, in its normalization in Education and palliative educational intervention [13]. Cortina [14] emphasizes that little by little innovative proposals are being made within the different educational centres, although this innovation is not accompanied by a great generic interest from the educator or from governmental bodies. Therefore, we see that there is still hard work to be done. The inclusion of death in education is still a pending transformation, a unique opportunity because although there are already some new proposals for the education of death, there is still a long way to go.

Rodríguez Herrero, Herrán and Cortina [8] explain how death has been and is a field of study accepted from different perspectives; however, it was not in education until very recently. These disciplines have focused their attention on death as a loss, social factor or suffering, defending that education can and should liberate its social and educational normalization as a possible building block for society with more mature values, cults, solidarity and humanity. Ramos [15] and Xinyi, Yifan, Shu and Fang [16] assume that education for death is neither a psychological intervention nor a teaching based on doctrines or beliefs; education on death is an applied pedagogy, a theory and formation that is built through death is to connect education with consciousness. It is a question of rethinking and questioning the meaning of what we do, assuming our own or others’ death. This is an emerging project that must be included in all formal and non-formal educational levels to give rise to the comprehensive human formation [17,18,19].

Education must help to contribute to knowledge and awareness of the existence of death, which is why we must work in different pedagogical ways (Education in values, emotional Education, Social Education and Health Education), but we cannot deny that Education for death is an issue under construction and that it does not have the same recognition and prominence in countries in which education is in constant transformation and development [20,21,22].

Education inclusion for death within a formal and non-formal educational system as a global, standardized and regular content is a part of the social education is to live fully. Dealing with death from the educational point of view can contribute to the development of a more aware, open and mature society that generate conditions conducive to promote the comprehensive growth of elderly [23,24,25]. Death education aims to develop pedagogical training both for teachers and students, from pre-school Education to higher education, adults and pensioners [21]. We cannot trust in that it is life itself that presents us with the facet of the process of dying but that education should also take its role being now more ready than ever [3,26,27].

Health education can provide a space for preventive intervention where different ways of conceptualising death can be explored, allowing them to address their own instability or anxiety, improving their conception, value system and integrating it as part of their life [28,29,30,31]. Through health education, older people can develop acceptance of the inevitable reality of death, overcoming feelings of despair or bitterness that are characteristic of their developmental stage [32,33]. Death education, from health education, helps to work towards physical, mental and social well-being, not just the absence of illness [34,35]. In addition, it involves helping emotional understanding, triggering reflections on the meaning of life and strengthening critical thinking and sharing of experiences [36,37]. The overall aim of this study is to find out what experience older people have had with death education and the importance they give to health education.

## 2. Materials and Methods

### 2.1. Study Design

The research is based on a qualitative approach, which allows us to describe, interpret and reflect on the information obtained through the research process [38,39]. The analysis of a social reality contextualised from the perspective of the elderly is proposed, developing a qualitative study of a descriptive and hermeneutic nature, characterised by a deductive-inductive process. The instrument used to collect information was a semi-structured interview designed ad hoc and validated through 10 expert judgement. Ethical approval for this study was granted by the Research Ethics Committee of the University of Granada (Spain).

These interviews were read by the interviewers and answered by each of the persons interviewed. In terms of the structure of the interview, this was divided into three parts: the first one collects the sociodemographic data of the person, age, sex, profession, marital status, address, telephone number, number of children and studies carried out, the second group collects the biographical data about the course of their life that influenced the subject’s memories (family, childhood, school, the usefulness of what was acquired, work, friends, retirement... and finally, the issues relating to the present, showing a special interest in the topic of death and preparation for the fourth age.

The interviews were carried out in the residence of the older adult during November 2019 and February 2020. The average interview time was two hours, organized in several sessions.

### 2.2. Participants

The participants in our study were 28 subjects (12 men and 16 women) from the province of Granada (Spain), aged between 61 and 78 years, most of them between 65 and 70 years old, the average age being 69 years, with a medium-high socio-cultural level, and who provided us with sufficient information to reach the theoretical saturation of the data (Table 1). The selection criteria were (a) geographical location in the rural or urban context of Granada (Spain), (b) age 60 years or older and (c) death of a close relative in the last 5 years. Once the informed consent for the study was signed, the interviews were conducted according to the schedule proposed by the participants, in private and quiet places for the informants, so that they could narrate their experiences in an uninterrupted manner and without distractions, with an approximate duration of 60 min. The semi-structured interview was designed on the basis of theoretical constructs, which are the result of the literature review and which respond to the research objective. The sequence of questions that make up the interview is divided into families of analysis, which in this study are education and preparation for death, analysing the importance they attach to health education, and which will respond to the need and importance of educating and preparing for death.

### 2.3. Data Analysis

The interviews were audio-recorded, with the prior consent of each of the interviewees, in order to subsequently, through the technique of content analysis, construct a system of deductive, inductive and emergent categories employing theoretical analysis and the information gathered by the participants using the Atlas.ti version 8 programme, in order to organise, reduce and analyse the information collected [40]. Similarly, a process of validation of the category system was carried out, which included the prior coding of five interviews by the researchers and four external specialists in the subject matter under study and in qualitative methodology in order to achieve reliability (Table 2).

### 2.4. Ethical Considerations

This research conforms to the ethical issues of the University of Granada. Prior to the interview, participants received instructions and an informed consent form, which included information about the aim of the study, process, method of data collection, rights, data handling and expected benefits of the research. The privacy of the participants and the place and time of the interviews was respected and the participants were not identified.

## 3. Results

**Figure 1:**
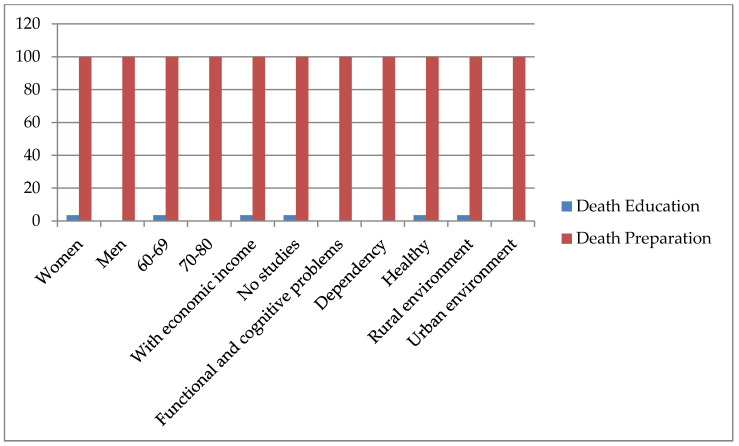
Death and preparation for death.

Generally, in the following figure (Figure 1), we can see the percentage of participants who have received an education for death and who describe the importance of receiving preparation for death. Only women, between 60 and 70 years of age, with no educational level and with an income who live in rural areas have received an education for death throughout their entire lives. However, all participants affirm the importance and necessity of receiving preparation for death.

**Figure 2:**
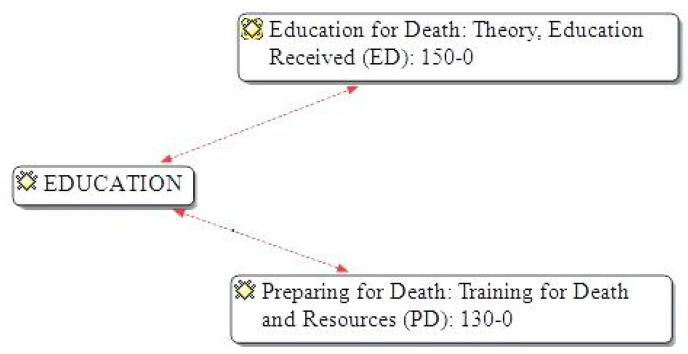
Semantic network Atlas.Ti.

The results have been organised according to the two categories of analysis (Figure 2): (1) education for death: theory, education received and (2) preparation for death: training for death and resources together with the importance they attach to health education.

In the following table (Table 3) we can see the most representative citations, indicating category and interviewee.

### 3.1. Category 1. Education for Death: Theory, Education Received

In Western society, the concept of death is a taboo subject. For this reason, when referring to the education on death received, we found that several of our informants (45%) had only received it in their childhood, under the influence of the Catholic religion and with a negative approach “no, never, apart from religion, I have not received anything” (I17-9:28), “no nothing. Religion taught me to fear death” (I-19-11:22), “as a child they would explain to me horrifying things about death concerning sins, purgatory, but everything from an educational point of view, and as a child, all this frightens you, makes you scared... they explained to us the phantasmagorical fact of death in which the dead would come if you didn’t behave well they would come and take you away” (I2-12: 21), “yes, they spoke to me in the Spiritual Exercises, then death was something very bad and they put fear in your body, death was something very terrible that made you worry about losing your place in paradise. It was part of religion, but if you were in sin, your life became bitter. You lived in terrible fear” (I16-14:16).

Similarly, during their lifetime, the majority of the participants (95%) claim not to have received any death education at any time. “No, I experienced the phenomenon but nobody explained anything about death to me” (I3-22:15), “never, nothing” (I9-28-29), recall having discussed the topic in the context of a funeral “no, it is a topic that is not talked about. I honestly believe that it is a topic that does not come up in a regular conversation with your loved ones or with your friends… unless at a funeral you talk about it concerning the person who has died. It’s not a subject that is talked about, it’s only talked about in those moments when it happens, has happened or is going to happen, and so we give it a bit more importance” (I20-30-45) or when someone is very close to death “you tend to remember death when you have it very present in you or in some of your relatives, when someone gets sick” (I5-26-23) or if one has reached an advanced age, “I talk about it with my friends, because they have elderly people in their care or they have suffered some experience recently and when it is painful it is a subject that is brought up, and especially at this age we are already thinking about how we are and what will happen. It’s mainly talked about because there is something wrong, but it’s not something you talk about naturally” (I12-4:16).

We can observe how over time, and especially nowadays, the subject of death is omitted from everyday life, making it difficult to conceive and elaborate on death and mourning, and this is why the promotion of health education should promote the teaching of death as part of life and educational accompaniment as a pedagogical basis in the mourning process. Not having received such training and education, most of them (95%) highlight the importance of the lived experience as a means of death education, “no. In my case, life is what has helped me “(I4-23-26), “never, just the experiences I have had” (I10-40-55), calling the experience education for life “specifically not for death, I have received an education for life and being a step towards death, all education for life should prepare for death, whether you are a believer being a step to the afterlife or being a non-believer as the end of our life trying to be happy filling you with peace and serenity” (I8-27-29).

### 3.2. Preparing for Death: Training for Death and Resources

This category encompasses two subcategories, training (importance of death preparedness as a means of promoting health education) and resources (how could death preparedness be carried out with older people? Informants (100%) state that this is a very important issue throughout life. “Yes, it is very important because it is part of life and therefore children, young people, adults and the elderly should be aware that death is there, even if it is being hidden. It should not be hidden from children and should be talked about and prepared for, seen as a natural process, help those who are in a process of deterioration and provide them with the care they need. Accept and do not hide. To face it as a natural process, to include it in the students’ contents, in the school...” (I1:1-41), and, as such, should begin to be worked on during childhood. “Yes, especially for children. My experience tells me that children have a greater strength than adults to absorb and comprehend things, they take things much better, without hiding anything, they can assimilate these processes” (I13:5-20). Increasingly, the need to educate for death from a very early age is being expressed, promoting a more intense, certain and closer attitude to life.

This is a very important issue at any stage of life. “We must bear in mind that the subject of death is extremely important at any stage of life, in young people who are so brave, sometimes without thinking about the existence of death, but they must also know the fragility of life, know the danger, for example, of traffic, we must know that this is a fact of human nature, we must be aware of the transience of life, the importance of time but that this is a sign of living each day as if it were the last, an invitation to live with intensity. Living as if it were the last” (I2-12:32), an education that is developed throughout the life cycle “a longitudinal education, from the time the child is born, but I must also say that the elderly have death constantly present in their lives even if they don’t say so, therefore it is also important to establish a strategy because people have a very hard time” (I10-2:26).

Death education has a function in which the emotional aspect or even a spiritual aspect can be worked on, for this reason, preparation for death is very important in hospitals, where death is very present, both for the family “of course, especially in different hospital proceedings. Sometimes when you are in the hospital during the death of a loved one you don’t know what to do and you are not taught how to deal with what is going to happen, just like when you have to confirm that a corpse of a loved one “is yours”, that process is mind-boggling and nobody prepares you for it” (I10:2-30), as for people close to death “when my friends were in a hospital, already quite ill, it would have been very useful to receive preparation for death by specialised professionals beforehand, especially for my best friend, as he did not want to die and lived that moment with a lot of suffering” (I25:30-50).

Preparation for death helps the development of healthy ageing through health education, as it is a means to promote the improvement of quality of life by eliminating and reducing the anxiety and stress it can cause. Numerous informants (98%) state that it is an education that brings life “it is about educating us for life, understanding that death is just another process of life, and as such, we should be prepared to live healthily and with quality of life” (I9:35-30), “We must not educate exclusively for death, we must educate for life and as such death is a part of life” (I20-10:30).

Finally, there was agreement that this is a difficult task due to the taboo cultural conception of death (100%), but that it is important to prepare for death by organising talks, workshops or courses “I think that talks or workshops should be organised to deal with this subject, so that they can be aware of it calmly, without fear or anxiety. To conceive death as something natural” (I35:8-45). Religion can help prepare believers “possibly, I stress the issue of religion for believers, it can help but from an agnostic point of view it would be prepared” (I1:1-46). Moreover, its inclusion in the university for adults “I remember once again when this class in the Permanent Open Training Classroom dealt with the subject of death, this teacher has a series of workshops to deal with the subject and I have involved many of my classmates so that they would sign up for these workshops” (I15: 7-25) through emotional or psychological education “emotional and psychological education could be an entry point concerning the topic” (I17-9:36), or by listening to other people’s experiences “experiences of people who have lived very close to death, this would help others to become aware and how to cope with it” (I21:14-31) as a specific health subject or as part of health promotion for elderly people.

In the training developed, the following contents can be worked on:

1. Delimitation and characterization of death: knowing the term itself from three perspectives (biological, psychological and social), cultural and religious aspects.
2. Grief: what is grief, phases or tasks of grief, coping strategies promote the teaching of death as part of life and educational accompaniment as a pedagogical basis in the grief process.
3. Health education as a means of addressing death: understand its definition, purpose and perspectives (palliative and preventive).

This proposal can be evaluated with the completion of discussion groups to find out what has been learned and aspects for improvement.

## 4. Discussion

The results of the study showed that most of the interviewees agree on the importance of this education, but in their life trajectory, it has only taken place during childhood and related to the religious sphere. Furthermore, they argue that work should start at a very early age to give a closer view of life and what death entails. These findings are consistent with previous studies [32,39,41,42,43] that claim that death education has a preventive function as it develops a conceptual and cognitive sense that involves the understanding of sub-concepts leading to the acceptance of biological death. The educational process can help to adapt to the new situation. McClatche [44], Ramos-Pla and Camats [45] and Martínez and Isidro de Pedro [46] assume that education for death is not about a psychological intervention and not one with teaching based on doctrines or beliefs; educating for death needs an applied pedagogy, a theory and training that is built through death to connect education with consciousness. It is important to work on the emotional aspect [47,48], as it can be a good opportunity to express feelings, concerns, fears, emotions, beliefs, etc., or even from a spiritual approach if one wants to find a meaning to existence or grief [49]. Death education enhances social support, spirituality and optimism through the implementation of specific and adaptive coping strategies to help overcome one’s own and others’ grief [50,51]. In Western culture, the subject of death is omitted, making it hard to mourn. For this reason, education for death should be a part of life and educational accompaniment as a pedagogical basis in the mourning process should be promoted [52].

The topic related to death has been removed from everyday life and replaced by specialised medical language, which is related to the tendency to avoid processes related to finitude [29]. The perspectives of the elderly on death confirmed that, in the field of education, it is still a taboo subject that tends to be ignored and pushed aside and as such, it takes on special relevance in the lives of our elders, as a way to face death itself in situations of anxiety and fear, as a simple accompaniment to overcome this process, they also expose the need for death education in the family environment, as a process of educational help on how to overcome the mourning of the deceased person. Death is seen as a taboo subject. Therefore, we must rethink this issue and promote an education for death that eliminates the non-approach of the subject as something natural, i.e., as part of our life cycle. As educators, we must learn to confront this issue and transmit and educate that death is part of the meaning and essence of life [18,21,52,53,54].

Preparation for health as a means to work on death must be included in the didactics. In this way, the purpose of this education is to reflect on the formation where death takes its natural place. Education is owed to knowledge and awareness about the existence of death; that is why we must work from different pedagogical lines (health education, education in values, emotional education and social education), but we cannot deny that education about death is a work in progress and does not have the same recognition and prominence in countries where education is in constant transformation and development [18,45,55,56]. The elderly show the need to develop a preparation for death through its inclusion as a cross-cutting health subject in schools, colleges and universities; development of courses and workshops focused on their own experiences, emotional education, preparation for their own and other people’s grieving, cinema-forums, lectures, etc. Therefore, it is once again important to include the topic of death education within health education, so that it is not only visualised but also linked to healthy ageing and quality of life for elderly people [57,58]. The inclusion of education for death within the formal and non-formal educational system as a global, normalized and ordinary content forms part of the teaching of living completely [48,59]. Our findings highlight the need to include it in the different subjects with which it is related or to create a specific one at the university for the elderly, as they relate how on certain occasions the subject has been dealt with from a historical or health point of view and the feeling of rejection and pain in the face of unresolved grief, or the fear of their own death, has been felt in class, and thus be able to develop a quality education for death. Numerous studies [3,13,28,60] concluded death takes place in the adaptation of the person at the psychological, physical, social and biological levels; studying the implications that this poses is of special relevance, implications that vary according to the current stage. Rodríguez Herrero, Herrán and Izuzquiza [26] together with Rodríguez Herrero, Herrán and Cortina [55] and Munar, Gaviria and Castañeda [61] and Kellehear [62] state that death education is an emerging area of knowledge based on the need to include topics in the formation of the person from their educational training.

The development of death education will improve people’s ability to reconstruct the meaning of their own lives through the confrontation with death. In this sense, education should focus on reducing existing fear and anxiety, showing awareness of the inevitability of death and integrating it into the life of the other [56,63,64,65].

Through the educational approach, we can implement educational actions to include death education in health education [32,43,45], through the previous or curricular approach working on the educational contents of the curricular areas but taking into account the transversal axes of education; elaborating from the idea of death; working with the presence of death; reasoning in a natural way; avoiding prejudices, patterns and schemes; and seeking reflection, self-criticism and transformation. It also offers a subsequent or palliative didactic approach, setting guidelines for the educational accompaniment developed under the process of accompaniment, specifically in two phases: pre-orientation phase, to avoid inappropriate self-questioning and to provide valid self-answers, not centred on one’s egocentrism but centred on one’s conscience, on the other and on a knowledge that can be assimilated to education for death, and orientation phase: based on the above information, it is possible to proceed through communicative empathy and the needs of the person being accompanied, based on provisions and guidelines: confidence in the educational accompaniment, knowledge of the situation and the person being accompanied, openness with ourselves to understand ourselves, clarity to be able to express ourselves, allowing them to express themselves. Here lies the importance of being able to create educational spaces for training and awareness in death [66,67,68].

## 5. Conclusions

Through the educational approach, we can implement educational actions to include education for death in health education. From the point of view of elderly people, it is important to develop a preparation for death through their inclusion as a cross-cutting health subject in schools, colleges and universities; development of courses and workshops focused on their own experiences; emotional education; preparation for their own and other people’s grieving; cinema-forums and lectures. From the promotion of health education, the teaching of death as part of life and educational support as a pedagogical basis in the grieving processes should be promoted.

Finally, the limitations of this study focus on the difficulty of dealing with the subject of death, especially with elderly people, as they are close to this final stage of life. Moreover, because of the sensitive topic of research, it is understandable if researcher does not openly discuss and share all their thoughts about the process. The results of this study are contextualised in the province of Granada and, despite the limitations, allow us to obtain information about death education in elderly people, as a means of favouring healthy ageing in the fourth stage of the life cycle. From these limitations, we can deduce the need for further research to be able to carry out an intervention programme for elderly people. In this study, the majority of participants have a healthy life, so it can limit the general vision of health education as a means to work on death in dependent people. Therefore, our next step would be to explore the views of dependent older people and those with functional and cognitive problems. Furthermore, due to local culture the findings and implications of this study may not be fully applicable to other countries.

## Figures and Tables

**Table 1:** Description of participants.

Gender		Aged		Educational Level		Income Level		Health			Geographic Location	
Women	Men	60–69	70–80	With studies	No studies	With economic income	No economic income	Functional and cognitive problems	Dependency	Healthy	Rural environment	Urban environment
42.85%	57.14%	60.71%	39.29%	100%	0%	100%	0%	10.71%	7.14%	82.14%	21.43%	78.57%

**Table 2:** Description of the analysis categories.

Category	Code	Definition
Education for death	ED	Theory and education received
Preparation for death	PD	Training for death and resources together with the importance they attach to health education

**Table 3:** Representative citations.

Category	Code	Referential Speech
(1) Education for death: theory, education received	ED	“Specifically, there is no subject of preparation for life or preparation for death, it is a set of many things, from school to family, friends or relationships with others along with work... it is a process very complex. It is about self-training” (I20-13: 23) (Informant 19, Document 9, Line 28)
(2) Preparation for death: training for death and resources together with the importance they attach to health education.	PD	“At all ages. Each age would require a different way, but taking charge of educating. To the children to know that it exists. To adults in the sense of accompanying. To the elderly person who is in the next process of dying, being in solidarity with our elderly and finally, to the person who has to die and who has to touch him at any time, he has to be prepared to face it whenever” (I1-1: 43).

## Data Availability

The data presented in this study are available on request from the corresponding author.

## References

[1] ReferencesGómez R. (2012). The doctor in the face of death (El médico frente a la muerte). Rev. Asoc. Española Neuropsiquiatría.

[2] Herrán A. (2020). The Pedagogy of Death in the Context of the Pandemic: A Radical and Inclusive Look (La Pedagogía de la muerte en el contexto de la pandemia: Una mirada radical e inclusive). Rev. Electrónica Educ..

[3] Testoni I., Biancalani G., Ronconi L., Varani S. (2019). Let's Start with the End: Bibliodrama in an Italian Death Education Course on Managing Fear of Death, Propensity for Fantasy and Alexithymia with a Mixed Method Analysis (Comencemos con el Final: BIBLIODRAMA en un Curso Italiano de Educación Sobre la Muerte Sobre el Manejo Del Miedo a la Muerte, la Propensión a la Fantasía y la Alexitimia con un Análisis de Método Mixto).

[4] Viguera V. (2006). Fears in Older Adults (Los Miedos en los Adultos Mayores). http://www.isalud.org/htm/pdf/pdfLazos/212LOS%20MIEDOS%20EN%20LOS.

[5] Casemiro F.G., Quirino D.M., Diniz M.A.A., Rodrigues R.A.P., Pavarini S.C.I., Gratão A.C.M. (2018). Effects of health education in the elderly with mild cognitive impairment. Rev. Bras. Enferm..

[6] Hyun E.M. (2014). Effect of death education program for university students. J. Korea Acad. Ind. Coop. Soc..

[7] Dadfar M., Farid A.A.A., Lester D., Vahid M.K.A., Birashk B. (2016). Effectiveness of death education program by methods of didactic, experiential, and 8A model on the reduction of death distress among nurses. Int. J. Med. Res. Health Sci..

[8] Gómez-Sancho M. (2010). End of Life Health Care: Concepts (Atención médica al final de la vida: Conceptos). Rev. Soc. Española Dolor.

[9] Martinez-Litago E., Martínez-Velasco M.C., Muniesa-Zaragozano M.P. (2017). Palliative care and end-of-life care in multipathological patients (Cuidados paliativos y atención al final de la vida en los pacientes pluripatológicos). Rev. Clínica Española.

[10] Sánchez-García M.R., Moreno-Rodríguez M., Hueso-Montoro C., Campos-Calderón C., Varella-Safont A., Montoya-Juárez R. (2017). Difficulties and favorable factors for care at the end of life in nursing homes: A study with focus groups (Dificultades y factores favorables para la atención al final de la vida en residencias de ancianos: Un estudio con grupos focales). Atención Primaria.

[11] Muñoz M.Z., Valladares M.P., Van-der Hofstadt Román C.J., González S.T., Portilla-Tamarit I., Rodríguez-Marín J. (2020). Satisfaction with hospital care at the end of life (Satisfacción con la atención hospitalaria al final de la vida). Rev. Clínica Española.

[12] Porcel-Gálvez A.M., Badanta B., Barrientos-Trigo S., Lima-Serrano M. (2021). Older people, dependency and vulnerability in the coronavirus pandemic: Emergence of social and health integration (Personas mayores, dependencia y vulnerabilidad en la pandemia por coronavirus: Emergencia de una integración social y sanitaria). Enfermería Clínica.

[13] Rodríguez Herrero P., Herrán A., Cortina M. (2015). Educate and Live with Death in Mind (Educar y Vivir Teniendo en Cuenta la Muerte).

[14] Cortina M. (2010). Cinema as a Didactic Resource for Education for Death: Training Implications for Teachers (El Cine como Recurso Didáctico de Educación para la Muerte: IMPLICACIONES Formativas Para el Profesorado). Ph.D. Thesis.

[15] Ramos R. (2010). Shooting stars do not grant wishes. Bereavement Prevention, Evaluation and Intervention Program in the School Context (Las Estrellas Fugaces no Conceden Deseos. Programa de Prevención, Evaluación e Intervención por Duelo en el Contexto Escolar).

[16] Xinyi Z., Yifan C., Shu L., Fang F. (2020). Death education in medical schools of the United States and its implications. Chin. J. Med. Educ..

[17] Lima A., Bergold M., Souza A., Barbosa M., Ferreira M. (2018). Education for death: Awareness for caring (Educación para la muerte: Sensibilización para el cuidar). Rev. Bras. De Enferm..

[18] Nan J.K.M., Pang K.S.Y., Lam K.K.F., Szeto M.M.L., Sin S.F.Y., So C.S.C. (2020). An expressive-arts-based life-death education program for the elderly: A qualitative study. Death Stud..

[19] Morgan J.D. (2020). Death education in the context of general education. Readings in Thanatology.

[20] Wang Y., Tang S., Hu X., Qin C., Khoshnood K., Sun M. (2020). Gender Differences in Attitudes Toward Death Among Chinese College Students and the Implications for Death Education Courses. Omega J. Death Dying.

[21] Farley G. (2018). Death anxiety and death education: A brief analysis of the key issues. Deliv. Cancer Palliat. Care Educ..

[22] Friesen H., Harrison J., Peters M., Epp D., McPherson N. (2020). Death education for children and young people in public schools. Int. J. Palliat. Nurs..

[23] Lim H., Kwang-Hwan K. (2019). A study on how elderly people are preparing for dying well. J. Korea Acad. Ind. Coop. Soc..

[24] Lee G., Hye-Jeong J., Jung-Ok Y. (2018). Factors Contributing to Death Preparation in Community-Dwelling Elderly: Using Korean National Survey on Elderly 2014. J. Korea Acad. Ind. Coop. Soc..

[25] Guner T.A., Zeynep E., Demir I. (2021). The Effect of Loneliness on Death Anxiety in the Elderly during the COVID-19 Pandemic. Omega J. Death Dying.

[26] Rodríguez Herrero P., Herrán A., Izuzquiza D. (2013). And if I die... where is my future? Towards an education for death in people with intellectual disabilities (Y si me muero… dónde está mi futuro Hacia una educación para la muerte en personas con discapacidad intellectual). Educ. XX1.

[27] Herrán A., Cortina M. (2007). Introduction to a pedagogy of death (Introducción a una pedagogía de la muerte). Educ. Y Future.

[28] McClatchey I.S., King S. (2015). The Impact of Death Education on Fear of Death and Death Anxiety Among Human Services Students (El impacto de la educación sobre la muerte en el miedo a la muerte y la ansiedad ante la muerte entre los estudiantes de servicios humanos). OMEGA.

[29] Doka K.J. (2015). Hannelore wass: Death education—an enduring legacy. Death Stud..

[30] Braun V., Clarke V. (2013). Successful Qualitative Research: A Practical Guide for Beginners (Investigación Cualitativa exitosa: Una guía Práctica para Principiantes.

[31] Kastenbaum R. (2012). Death, Society and Human Experience (Muerte, Sociedad y Experiencia Humana).

[32] Testoni I., Tronca E., Biancalani G., Ronconi L., Calapai G. (2020). Beyond the wall: Death education at middle school as suicide prevention. Int. J. Environ. Res. Public Health.

[33] Khademi F., Siamak M., Mohamad G. (2020). The COVID-19 pandemic and death anxiety in the elderly. Int. J. Ment. Health Nurs..

[34] World Health Organisation (2015). Informe Mundial Sobre el Envejecimiento y la Salud. https://www.who.int/ageing/publications/world-report-2015/es/.

[35] Menzies R.E., Zuccala M., Sharpe L., Dar-Nimrod I. (2018). The effects of psychosocial interventions on death anxiety: A meta-analysis and systematic review of randomised controlled trials. J. Anxiety Disord..

[36] Testoni I. (2016). Psychology of grief and morire: Dal lavoro clinico alla death education (Psicologia del lutto e del morire: Dal lavoro clinico alla death education). Psicoter. Sci. Um..

[37] Hoeksema A.R., Peters L.L., Raghoebar G.M., Meijer H.J., Vissink A., Visser A. (2017). Oral health status and need for oral care of care-dependent indwelling elderly: From admission to death. Clin. Oral Investig..

[38] Sutton J., Austin Z. (2015). Qualitative Research: Data Collection, Analysis, and Management. Can. J. Hosp. Pharm..

[39] Chou H.J., Tseng K.Y. (2020). The experience of emergency nurses caring for patients with mental illness: A qualitative study. Int. J. Environ. Res. Public Health.

[40] Muhr T. (1991). ATLAS/ti—A prototype for the support of text interpretation. Qual. Sociol..

[41] Wang L., Yang L., Di X., Dai X. (2020). Family Support, Multidimensional Health, and Living Satisfaction among the Elderly: A Case from Shaanxi Province, China. Int. J. Environ. Res. Public Health.

[42] Testoni I., Bisceglie D., Ronconi L., Pergher V., Facco E. (2018). Ambivalent trust and ontological representations of death as latent factors of religiosity. (Confianza ambivalente y representaciones ontológicas de la muerte como factores latentes de la religiosidad). Cogent Psychol..

[43] Rodríguez Herrero P., De la Torre G. (2012). Education for Death and Grief in People with Intellectual Disabilities (Educación para la Muerte y el Duelo en Personas con Discapacidad Intellectual). https://s3.amazonaws.com/academia.edu.documents/46434395/EDUCACION_PARA_LA_MUERTE_Y_EL_DUELO_EN_PERSONAS_CON_DISCAPACIDAD_INTELECTUAL.pdf.

[44] McClatchey I.S., King S. (2015). The impact of death education on fear of death and death anxiety among human services students. Omega J. Death Dying.

[45] Ramos-Pla A., Camats R., Guàrdia R. (2018). Foundations for a preventive pedagogy on death at school (Fundamentos para una pedagogía preventiva sobre la muerte en la escuela). Rev. Complut. Educ..

[46] Martínez M., Isidro de Pedro A.I. (2020). Education for death and dying for a full life: Learning to die in order to learn to live (Educación para la muerte y el morir para una vida plena: Aprender a morir para aprender a vivir). Rev. Infad Psicol. Int. J. Dev. Educ. Psychol..

[47] Santiesteban J.R., Chang J.Z., Porozo C.H.A., Campoverde D.A.G., Santillán M.A.B., Palma P.D.R.C. (2018). Approach to the death process at school (Abordaje del proceso de la muerte en la escuela). Rev. Cuba. Investig. Biomédicas.

[48] Santiesteban J.R.G., Chang J.Z., Porozo C.H.A., Campoverde D.A.G., Santillán M.A.B., Palma P.D.R.C. (2018). Emotional education. Approach to the process of death at school. Rev. Cuba. Investig. Biomédicas.

[49] Asgari Z., Naghavi A. (2020). Teenage Post-traumatic Growing Experience after Sudden Loss of Father (Experiencia del crecimiento postraumático de adolescentes después de la pérdida repentina del padre). Loss Trauma.

[50] Roepke A.M. (2015). Psychosocial interventions and post-traumatic growth: A meta-analysis (Intervenciones psicosociales y crecimiento postraumático: Un metaanálisis). Clin. Psychol..

[51] Meichsner F., O'Connor M., Skritskaya N., Shear M.K. (2020). Grief before and after bereavement in the elderly: An approach to care. Am. J. Geriatr. Psychiatry.

[52] Grau J., Chacón M. (2002). Death and Attitudes to Death: A Review (La Muerte y las Actitudes Ante la Muerte: Una Revision).

[53] Cacciatore J., Thieleman K., Killian M., Tavasolli K. (2015). Braving human suffering: Death education and its relationship to empathy and mindfulness. Soc. Work Educ..

[54] Kim S.H. (2015). A meta-analysis of effectiveness of death education. Korean J. Hosp. Palliat. Care.

[55] Rodríguez Herrero P., Herrán A., Cortina M. (2019). International antecedents of the Pedagogy of death (Antecedentes internacionales de la Pedagogía de la muerte). De Educ..

[56] Ramos-Pla A., Gairín J., Camats R. (2018). Practical and functional principles in situations of death and bereavement for education professionals (Principios prácticos y funcionales en situaciones de muerte y duelo para profesionales de la educación). Reice. Rev. Iberoam. Sobre Calid. Efic. Cambio Educ..

[57] Solaimanizadeh F., Mohammadinia N., Solaimanizadeh L. (2020). The relationship between spiritual health and religious coping with death anxiety in the elderly. J. Relig. Health.

[58] Linjia X., Shuang C., Nan A. (2015). An Analysis of the Hospice Care and Death Education in the Health Communication Field. Journalism.

[59] Barry M.M. (2000). Death education: Knowledge, attitudes, and perspectives of Irish parents and teachers. Death Stud..

[60] Martínez-Heredia N. (2020). Análisis cienciométrico de mayor impacto acerca del duelo y la pérdida en personas con discapacidad intellectual. Siglo Cero.

[61] Munar Ó.O., Gaviria E.P., Castañeda D.A.O. (2020). Pedagogy of death at school: A pending task (Pedagogía de la muerte en la escuela: Una tarea pendiente). Rev. Educ. Ciudad.

[62] Kellehear A. (2015). Death education as a public health issue. Death Dying Bereave. Contemp. Perspect. Inst. Pract..

[63] Pedrero-García E. (2019). Education for Health and the pedagogy of death: Perceptions and demands of university teachers in Spain (Educación para la Salud y pedagogía de la muerte: Percepciones y demandas del profesorado universitario en España). Interface Comun. Saúde Educ..

[64] Rodríguez Herrero P., Herrán A., Cortina M. (2012). Background of the pedagogy of death in Spain (Antecedentes de la pedagogía de la muerte en España). Enseñanza Y Teach..

[65] Kim S.H., Byun S.W. (2014). Analysis of the trends of research education on death. J. Digit. Converg..

[66] Nienaber K., Goedereis E. (2015). Death anxiety and education: A comparison among undergraduate and graduate students. Death Stud..

[67] Rodríguez Herrero P., Herrán Gascón A., Miguel Yubero V. (2020). The inclusion of death in the curriculum of the Spanish Regions. Comp. J. Comp. Int. Educ..

[68] Corr C.A. (2016). Teaching about life and living in courses on death and dying. Omega J. Death Dying.

